# Outcome of MS relapses in the era of disease-modifying therapy

**DOI:** 10.1186/s12883-017-0927-x

**Published:** 2017-08-07

**Authors:** Muriel Stoppe, Maria Busch, Luise Krizek, Florian Then Bergh

**Affiliations:** 1 Department of Neurology, University of Leipzig, Liebigstraße 20, 04103 Leipzig, Germany; 2 Translational Centre for Regenerative Medicine, University of Leipzig, Liebigstraße 20, 04103 Leipzig, Germany

**Keywords:** Multiple Sclerosis, Relapse, Relapse treatment, Relapse management, Relapse outcome, Methylprednisolone, Immunoadsorption, Plasmapheresis, Prospective study

## Abstract

**Background:**

In multiple sclerosis (MS), neurological disability results from incomplete remission of relapses and from relapse-independent progression. Intravenous high dose methylprednisolone (IVMP) is the established standard treatment to accelerate clinical relapse remission, although some patients do not respond. Most studies of relapse treatment have been performed when few patients received disease-modifying treatment and may no longer apply today.

**Methods:**

We prospectively assessed, over one year, the course of patients who presented with a clinically isolated syndrome (CIS) or MS relapse, documenting demographic, clinical, treatment and outcome data. A standardized follow-up examination was performed 10–14 days after end of relapse treatment.

**Results:**

We documented 119 relapses in 108 patients (31 CIS, 77 MS). 114 relapses were treated with IVMP resulting in full remission (29.2%), partial remission (38.7%), no change (18.2%) or worsening (4.4%). In 27 relapses (22.7%), escalating relapse treatment was indicated, and performed in 24, using double-dose IVMP (*n* = 18), plasmapheresis (*n* = 2) or immunoadsorption (*n* = 4).

**Conclusions:**

Standardised follow-up visits and outcome documentation in treated relapses led to escalating relapse treatment in every fifth relapse. We recommend incorporating scheduled follow-up visits into routine relapse management. Our data facilitate the design of prospective trials addressing methods and timelines of relapse treatment.

## Background

Multiple sclerosis (MS) is the most common non-traumatic disease that causes permanent neurological deficits in young adults. It is a progressive, autoimmune disorder of the central nervous system (CNS), characterized by inflammatory lesions and demyelination which result in injury of myelin sheaths, oligodendrocytes, and of axons as well as entire neurons [1, 2]. Neurological disability results from accumulating residual deficits of acute MS relapses throughout the individual’s disease course, and from insidious progression at later stages. Although MS relapses can spontaneously recover, several studies proved superior clinical outcome with high dose intravenous methylprednisolone (IVMP) treatment [3–6]. However, duration and degree of recovery of acute MS attack vary not only interindividually but also intraindividually over the course of the disease. Some relapses do not fully remit despite treatment. The data quantifying clinical outcome of MS relapses are based on randomised trials which were performed before availability and common use of disease-modifying therapy (DMT) [7–11], and may no longer reflect clinical reality. Moreover, escalating relapse treatment has become more common. Besides the application of a second course of high dose IVMP, extracorporeal procedures such as plasma exchange (PLEX) and immunoadsorption (IA) are increasingly used in steroid-resistant MS relapses [12–17]. Nevertheless, due to the lack of comparative studies, there is no standard approach for indication and employment of escalating relapse treatment in ongoing relapse. Evaluating how well severity and duration of the exacerbation are improved represents the most valuable and clinically meaningful assessment in determining the efficacy of relapse treatment. Thus, data investigating the clinical outcome in acute MS relapses in the era of wide-spread use of DMT needs to be collected and analysed systematically.

## Methods

We prospectively assessed, over one year, data of patients who presented to the Department of Neurology at the University of Leipzig with either a relapse of established MS or clinically isolated syndrome (CIS) of CNS demyelination, including both outpatients and inpatients. Based on prior approval by the University of Leipzig’s Ethics Committee, patients are requested, upon admission to our hospital, to consent to statistical analyses of anonymous diagnostic and treatment information for scientific and quality assurance purposes. We included only anonymous data from patients who consented to this request. To facilitate systematic analysis, we developed a documentation sheet to collect demographic data, MS history (onset, disease-modifying therapy (DMT), latest neurological evaluation before current relapse), characteristics of the current relapse (symptoms, Kurtzke’s Expanded Disability Status Scale (EDSS) and Functional Systems Scale (FS), visual acuity (VA), primarily affected FS, date of relapse onset) and treatment of current relapse (drug, dose, application route, duration). A standardized follow-up examination was performed and documented in the Neuroimmunology outpatient clinic after primary relapse treatment in order to evaluate the clinical outcome and to decide whether an escalation of treatment was necessary; if escalation treatment was performed, an equivalent follow-up visit was performed after each treatment cycle. History was taken and examination performed by physicians experienced in evaluation of MS patients, including former neurostatus training and certification (www.neurostatus-systems.net). Recovery was defined based on both subjective symptoms and objective findings on neurological examination related to the current relapse (scored by Kurtzke Functional System and EDSS ratings). “Complete recovery” denotes complete resolution of symptoms and a neurological examination as documented pre-relapse (or, in first episodes, a normal neurological examination, EDSS 0). Accordingly, “partial response” refers to improvement in symptoms or/and FS score not returning to pre-relapse score, “no response” to unchanged symptoms and neurological findings, and “worsening” to an increase in the FS score relevant to the current relapse (which was always paralleled by an increase in symptoms). Descriptive statistics was calculated as indicated in the text and tables. Data are presented as mean ± standard deviation (SD) unless indicated otherwise. To analyse potential differences in the outcome of relapse treatment in different patient groups, we used Fisher’s exact test (SPSS 11, SPSS Inc.).

## Results

**Fig. 1:**
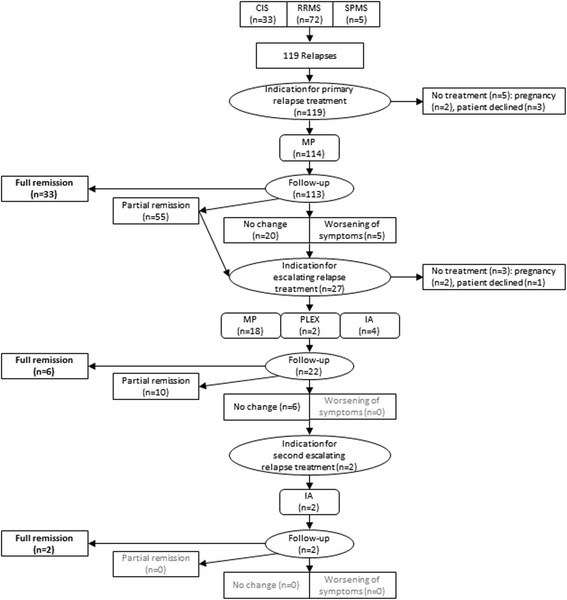
Flow chart of the patients and documented relapses. CIS = clinically isolated syndrome. IA = immunoadsorption. MP = methylprednisolone. PLEX = plasma exchange. RRMS = relapse-remitting multiple sclerosis. SMPS = secondary-progressive multiple sclerosis

Overall, we documented 119 acute relapses in 108 patients (Table 1, Fig. 1). The average age of the 73 women (67.6%) and 35 men (32.4%) was 34.7 ± 9.7 years. Of these 108 patients, 31 presented with CIS, 72 with relapsing-remitting MS (RRMS) and 5 patients with a relapse during secondary-progressive MS (SPMS).

**Table 1:** Demographic characteristics

Sex, n (%)	
Female	73 (67.6)
Male	35 (32.4)
Age at relapse onset (years)	34.7 ± 9.7
Clinical course, n (%)	
CIS	31 (28.7)
RRMS	72 (66.7)
SPMS	5 (4.6)
DMT in RRMS/SPMS, n (%)	
DMT	41/77 (53.2)
No DMT	36/77 (46.8)
DMT distribution, n (%)	
Interferon beta	15 (36.6)
Glatiramer acetate	9 (22.0)
Fingolimod	6 (14.6)
Natalizumab	4 (9.8)
B-cell depleting antibody^a^	2 (4.9)
Dimethyl fumarate	1 (2.4)
Teriflunomide	1 (2.4)
Mitoxantrone	1 (2.4)
Interferon + Teriflunomid^a^	1 (2.4)
Methylprednisolone^b^	1 (2.4)
DMT duration (years)	2.1 ± 2.5
Relapses, n	119

*CIS* clinically isolated syndrome, *DMT* disease-modifying therapy, *RRMS* relapse-remitting multiple sclerosis, *SMPS* secondary-progressive multiple sclerosis. ^a^ = within clinical trial. ^b^ = monthly methylprednisolone as individual approach

In the 77 patients with established MS, the disease was diagnosed 5.7 ± 15.4 years before current relapse; 41 patients (53.2%) had received a DMT for 2.10 ± 2.45 years: Interferon beta in 15 patients (36.6%), glatiramer acetate in 9 patients (22.0%), fingolimod in 6 patients (14.4%), natalizumab in 4 patients (9.8%), a B-cell depleting antibody in 2 patients within clinical trial (4.9%), teriflunomide, interferon beta plus teriflunomide within a clinical trial, dimethyl fumarate, mitoxantrone or monthly methylprednisolone in one patient each (2.4%). For the 88 relapses observed in established MS, the mean EDSS before relapse was 2.2 ± 2.0, while prior EDSS was unknown in 6 cases.

The 31 patients presenting with CIS did not receive DMT. EDSS before symptom onset was, of course, unavailable and was defined as zero.

One hundred fourteen of the 119 documented relapses (95.8%) were treated, all with high dose IVMP with an average of 3531.6 mg ± 1164.3 mg over 3.6 ± 1.0 days (985 ± 151 mg/d). Treatment was initiated 13.8 ± 19.4 days after the onset of relapse symptoms. For all treated relapses (CIS, RRMS, and SPMS, *n* = 114), the mean onset EDSS was 3.1 ± 1.6 and improved to 2.6 ± 1.8 after primary relapse treatment. In MS relapses (*n* = 88), the mean EDSS at relapse onset was 3.5 ± 1.6 and improved to 3.0 ± 1.8 after primary relapse treatment. In CIS (*n* = 31), the mean EDSS at relapse onset was 2.2 ± 1.0 and improved to 1.4 ± 1.1 after primary relapse treatment. Mainly affected FS in all relapses (*n* = 119) were sensory (*n* = 51, 42.9%), motor (*n* = 35, 29.4%) and visual system (*n* = 29, 24.4%), followed by brainstem, cerebellum, ambulation, bladder function and cognition. In 38 relapses (31.9%), more than one FS was affected (Table 2). In 47 relapses (39.5%), symptoms occurred in a previously affected FS. For the remaining 72 relapses (60.5%), the affected FS was zero before symptom onset (CIS: *n* = 31, 100%; RRMS: *n* = 40, 43.1%; SPMS: *n* = 1, 1.3%).

**Table 2:** Affected functional systems in all documented relapses (*n* = 119)

Affected FS in all relapses, n (%)	
Sensory	51 (42.0)
Motor	35 (29.4)
Visual	29 (24.4)
Brainstem	20 (16.8)
Cerebellum	15 (12.6)
Ambulation	14 (11.8)
Bowel and bladder	7 (5.9)
Cerebral	1 (0.8)
Relapses with more than one affected FS, n (%)	38 (31.9)

*FS* functional system

For CIS only (*n* = 31), mainly affected FS were visual (*n* = 15, 48.8%), sensory (*n* = 7, 22.6%), brainstem (*n* = 6, 19.4%) and motor system (*n* = 5, 16.1%), followed by cerebellum and cognition. Bladder function and ambulation were not affected in CIS. In 4 CIS relapses (12.9%), more than one FS was affected (Table 3).

**Table 3:** Affected functional systems in clinically isolated syndrome (*n* = 31)

Affected FS in CIS, n (%)	
Visual	15 (48.4)
Sensory	7 (22.6)
Brainstem	6 (19.4)
Motor	5 (16.1)
Cerebellum	3 (9.7)
Cognition	1 (3.2)
Ambulation	0 (0)
Bowel and bladder	0 (0)
Relapses with more than one affected FS, n (%)	4 (12.9)

*CIS* clinically isolated syndrome *FS* functional system

In 23 relapses (19.3%), the symptoms were reflected by an increase in the FS score, while the overall EDSS remained unchanged. In 16 relapses (13.4%), there was neither a change in FS nor in EDSS score (as compared to the latest available examination).

Follow-up examination was scheduled 10 to 14 days after the end of primary relapse treatment to evaluate clinical outcome and the indication for escalating relapse treatment. Of all 114 treated relapses, the follow-up visit was done in all but one, at a median of 14 days (interquartile range 7–38 days) after end of primary relapse treatment. In 88 relapses (77.9%), follow-up occurred within 42 days allowing for the initiation of escalation steps. In the remaining 25 relapses, patients declined an extra visit, and follow-up took place during regular appointment within a 3-to-6-months interval.

**Fig. 2:**
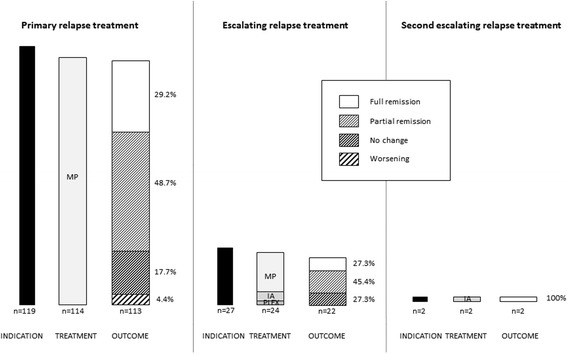
Allocation and effects of therapies. For primary *(left*), escalating (*center*) and second escalating (*right*) relapse treatment, the solid bar indicates the number of patients with indication for relapse treatment, the grey-shaded bar indicates applied treatment type, and the hatched bar indicates the effect of treatment, assessed clinically 10–14 days after treatment conclusion; the percentages of treatment response is given to the right of the hatched bar. IA, immunoadsorption; MP, methylprednisolone i.v.; PLEX, plasma exchange

Follow-up of relapses treated with primary relapse treatment (Fig. 2) revealed full remission (*n* = 33, 29.2%), partial remission (*n* = 55, 48.7%), no change (*n* = 20, 17.7%) or worsening of symptoms (*n* = 5, 4.4%).

In 27 of all 119 relapses (22.7%) and 113 follow-up visits (23.9%), escalating relapse treatment was indicated. In these relapses, primarily affected functional systems were visual (*n* = 11, 40.7%), motor (*n* = 7, 25.9%) or sensory (*n* = 4, 14.8%). Escalating relapse treatment was performed by a second course of IVMP (*n* = 18, 66.7%) treated with 3866.5 mg ± 2832.1 mg for 3.9 ± 1.1 days (1528 ± 539 mg/d), plasma exchange (5 sessions, *n* = 2, 7.4%) or immunoadsorption (5 sessions, *n* = 4, 14.8%). In the remaining three relapses (11.1%), further escalation was indicated but not performed due to pregnancy (*n* = 2, 7.4%) or decline by the patient (*n* = 1, 3.7%). Treatment with escalating relapse treatment (*n* = 24, but loss of follow-up in 2 patients) yielded full remission (*n* = 6, 27,3%), partial remission (*n* = 10, 45.4%) or no change (*n* = 6, 27,3%).

In two relapses, the indication for a second escalation step was confirmed. In both relapses, primary and escalating relapse treatment was performed with IVMP but with no change of relapse symptoms (loss of visual acuity or ataxia). Second escalation relapse treatment was performed as immunoadsorption (5 cycles) in both relapses and resulted in full recovery.

In the relapses manifesting as optic neuritis (ON, *n* = 29), the mean visual acuity (VA) of the affected eye before relapse was 0.97 ± 0.10. Except for one patient (VA 0.9), all ON were treated with primary relapse treatment (i.e. first-time IVMP) with VA amelioration from 0.40 ± 0.25 in acute relapse (*n* = 28) to 0.62 ± 0.33 (*n* = 27, one loss of follow-up). In 11 ON, indication for escalating relapse treatment was confirmed. Due to pregnancy in one ON, only 10 ON were treated with escalating relapse treatment and VA improved from 0.33 ± 0.16 at ON onset and 0.47 ± 0.31 after primary treatment to 0.58 ± 0.30. One of these ON was treated with a second escalating relapse treatment and VA recovered from 0.33 at ON onset and 0.33 after primary treatment to 0.70; this constituted full recovery to the latest pre-ON VA (Table 4).

**Table 4:** Visual acuity in all ON that received primary treatment (*n* = 28)

	Number	Visual acuity
Before ON onset	28	0.97 ± 0.11
At ON onset	28	0.40 ± 0.25
After primary relapse treatment		
All	27^a^	0.62 ± 0.33
Indication for escalating relapse treatment	11	0.47 ± 0.31
Escalating relapse treatment performed	10	0.47 ± 0.30
After escalating relapse treatment		
All	10	0.58 ± 0.33
Indication for second escalating relapse treatment	1	0.33
Escalating relapse treatment performed	1	0.33
After second escalating relapse treatment	1	0.70

*ON* optic neuritis ^a^ = one loss of follow-up

Forty five relapses (37.8% of all relapses, 51.1% of relapses in established MS) occurred under ongoing DMT with full remission in 18 relapses (40.0%), partial remission in 19 relapses (42.2%), no change in 7 relapses (15.6%) and worsening in 1 relapse (2.2%) after primary relapse treatment. In MS relapses without DMT, outcome after primary relapse treatment was full remission in 9 (20.9%), partial remission in 22 (51.2%), no change in 10 (23.3%) and worsening and loss of follow-up in 1 relapse (2.3%) each. This suggestive trend did not reach statistical significance for outcome of MS relapses under ongoing DMT vs. relapse outcome without DMT (Fisher’s exact test, *p* = 0.169). Outcome of CIS attacks (all without DMT) was full remission in 10 (32.3%), partial remission in 15 (48.4%), no change in 3 (9.7%) and worsening in 3 attacks (9.7%) after primary treatment. Adding all 74 relapses (62.2%) in patients who were either therapy-naïve or had stopped former DMT, outcome was full remission in 19 (25.7%), partial remission in 37 (50.0%), no change in 13 (17.6%), worsening in 4 (5.4%) and 1 relapse with unknown outcome (1.4%) after primary relapse treatment. Of the 27 relapses where indication for escalating treatment was raised, 10 occurred under ongoing DMT and 17 in therapy–naïve patients (7 CIS and 10 MS).

Either full remission or improvement by at least one full EDSS point occurred at the first follow-up visit in 48.4% of all patients with EDSS-relevant relapses (65.5% of CIS patients and 40.6% of patients with established MS, respectively). When differentiating outcome according to DMT, the same criteria for treatment response were met by 50.0% of patients with established MS on DMT and 32.0% of those without DMT (Fisher’s exact test, *p* = 0.119).

To address the question of optimal timing of the follow-up visit, we analysed outcome and treatment decision in the patients seen early (within 14 days) or later (15–42 days) after primary relapse treatment, based on the median time to follow-up. This revealed full remission in 24 and 28%, partial remission in 46 and 43%, no response in 19 and 23%, and worsening in 8.7 and 2.3%, respectively. Escalation treatment was indicated in 24 and 21%, respectively.

## Discussion

High-dose intravenous methylprednisolone (IVMP) is the established treatment for relapses in MS to accelerate the remission of relapse symptoms [18]. However, there are different regimes concerning dosage and form of administration of methylprednisolone (MP), and variable recommendations as to the interval within which therapy should be initiated. Oral MP appears to be equally efficacious as IVMP at equivalent doses [7, 8, 19, 20], whereas higher doses of IVMP have been proven to be more efficient than lower doses, concerning both clinical outcome and reduction of contrast-enhancing lesions on MRI [21]. Experimental data also support higher doses [22]. Regardless of the variants of MP treatment, only a fraction of treated patients fully recover from their symptoms. According to current guidelines, this clinical problem is dealt with by administration of a second course of IVMP at a higher dose, and eventually plasma exchange or immunoadsorption [18, 23, 24].

In our sample, 37.8% of the observed relapses (51.1% of all RRMS/SPMS-patients) occurred under DMT, while the remaining relapses were either initial presentations (CIS) or occurred in MS patients off DMT. This limits the conclusions we can draw regarding relapses during ongoing DMT, one of our primary goals. Interestingly, however, success of primary relapse therapy, defined as full remission of relapse symptoms was more often observed in patients with ongoing DMT (40.0%) than in those without DMT (26.0%). Together with the lower number of relapses on DMT, this supports a firm therapy attitude as demanded in current guidelines [18, 24].

Previous studies show remarkably variable response rates (improvement of at least 1 EDSS point) between 50 and 80% after 28 days [7, 8, 19, 20]. All four studies were designed to compare oral with intravenous methylprednisolone in patients with acute relapse but only including patients with clinically definite MS [7, 8] or fulfilling the 2005 McDonald criteria [19, 20], whereas we included all relapses in CIS, RRMS and SPMS. Alam et al. [7] report 20 patients receiving 500 mg IVMP for 5 days of whom 80% improved, from a mean Kurtzke disability score of 4.85 at baseline to 3.5 at day 28. Barnes et al. [8] report 36 patients receiving 1000 mg IVMP for 3 days, with 18 patients (50%) improving after 4 weeks on the pyramidal FS (not mentioning the absolute value of improvement) and over all patients, an improvement of a mean of 0.5 points in the EDSS. The more recent work of Ramo-Tello et al. [19] and Le Page et al. [20] report an improvement of at least one EDSS point after 28 days in 65% of 23 patients and 80% of 90 patients, respectively. However, full recovery was reached only in 40% [20]. Despite the different follow-up schedule, our response rates after 10–14 days (48.4% full remission or EDSS improvement) are consistent with the additionally reported 39% after 7 days of Ramo-Tello et al. [19] but differ from Barnes et al. who report no improvement of EDSS after 1 week. However, Barnes et al. do not describe their cohort in terms of DMT but have excluded patients on immunosuppressants. Since the study was published in 1997, we suppose DMT rates to be low. Ramo-Tello et al. state a DMT rate of 56.4%, comparable to our cohort.

Relating to DMT, our data suggest a trend to better steroid-responsiveness in MS under DMT than without DMT. However, comparing more recent data from cohorts with 55–56.4% of patients under DMT [19, 20] and our current data on the one hand, to results of older studies [7, 8], response rates are still comparable. As to average EDSS scores at relapse onset, our data are comparable with the recent studies [19, 20], whereas the EDSS in the older cohorts is clearly higher [7, 8]. As observed in a standardized follow-up examination, we indicated escalating relapse treatment in 22.7% of all relapses (23.9% of all follow-up visits) due to insufficient improvement after primary relapse treatment. Scheduled follow-up visit and detailed outcome documentation including EDSS after primary relapse treatment appears to lead to a fairly high rate of escalations. Unfortunately, data for relapse outcome without the explicit intention of follow-up are unavailable (and for systematic reasons may remain impossible to acquire). Also, our cohort is presumably not entirely comparable with relapses that present to neurological practitioners: we may have observed more severe relapses, given that we collected our sample from an outpatient clinic linked to a university hospital. This possible bias may limit the generalizability of our conclusion while, however, focussing the observation on the subgroup of MS patients who require the most efficient relapse treatment. In either case, integrating a follow-up visit 10 to 14 days after the end of relapse treatment into the routine of medical MS management, rather than relying on patients to return if unsatisfied, will – in our opinion - raise patients’ expectations towards improvement of relapse symptoms, and encourage them to ask for escalating relapse treatment. At the same time, this will apply to treating neurologists’ expectations as well, and scheduled follow-up should improve treatment quality. This is exemplified by (admittedly rare) relapses with irrelevant improvement after primary and escalating relapse treatment, in which full recovery emerged after second escalating relapse treatment. The need for scheduled follow-up visits is also supported by our observation that primary treatment achieves full recovery in less than one-third of relapses. With respect to optimal timing of the follow-up visit, longer follow-up (2–6 weeks) yielded a slightly higher proportion of “full recovery” while “worsening” was stated more often when follow-up was shorter (up to 2 weeks). Interestingly, however, the indication for escalating relapse treatment was confirmed in a remarkably similar proportion. Thus, patients recovering, but also those requiring escalation treatment can be identified early after primary relapse treatment. Taking into consideration that any relapse treatment is probably most successful within 6 weeks after symptom onset, and given that some patients require second escalation treatment, this argues for a follow-up visit at around two weeks after primary treatment, to allow for an equivalent period to evaluate the effect of escalated therapy. On the other hand, integrating relapse-specific documentation and follow-up visits into medical routine challenges clinic capacity, requires networking between in-patient and out-patient departments to organize escalating relapse treatment and schedule follow-up visits, flexible clinic schedules, and compliant patients.

Evaluation of the relative effectiveness of therapies in relapse treatment requires valid outcome measures that reflect the initial aggravation and subsequent improvement of the patients’ symptoms in relapses [25]. We observed that in 23 relapses (19.3% of all relapses), clinical aggravation was reflected in at least one FS but not the integrated EDSS, and in 16 relapses (13.5% of all relapses) both values remained unchanged. This confirms that the indication for treatment of an acute MS-relapse should not be based solely on changes in FS or EDSS but always integrate the impact upon the individual patient’s daily activities. At the same time, this shortcoming should not discourage neurologists from documenting relapse severity using quantitative scales. This is certainly best achieved in optic neuritis, and decline of visual acuity accounted for one quarter of MS relapses and almost half of CIS cases in our series. Importantly, visual acuity should be investigated regardless of reported relapse symptoms, since it revealed an additional affected FS in almost one-third of this cohort, increasing the sensitivity of the follow-up investigation. The mechanisms of action of IVMP include a wide range of effects on the immune system; DMTs may impact these effects in different ways [26, 27].

In the recent past, different possibilities of treatment for acute MS-relapses were explored, proposing plasmapheresis and immunoadsorption as alternatives to oral or intravenous MP either for steroid unresponsive MS relapses [13, 15] or when primary application of IVMP is not suitable (e.g. during pregnancy, MP intolerance, MP allergy) [18, 28, 29]. So far, these treatments are based on retrospective case series, and controlled trials to establish their risk-benefit ratio are not available. In order to design such prospective clinical trials to investigate the relative effectiveness of the different relapse therapies, our data provide a basis for estimating effects and derive required sample sizes.

## Conclusion

Intravenous methylprednisolone continues to improve relapse outcome. While its effect appears superior in patients with concomitant disease-modifying treatment, this effect may be confounded by a trend toward less severe relapses in the era of DMT. Nevertheless, we recommend scheduled follow-up visit and detailed outcome documentation after primary relapse treatment in order to identify the fairly high number of patients whose outcome can be further improved by employing escalating relapse treatment.

## Abbreviations

CIS: Clinically isolated syndrome
CNS: Central nervous system
DMT: Disease-modifying therapy
EDSS: Expanded disability status scale
FS: Functional systems scale
IA: Immunoadsorption
IVMP: Intravenous high dose methylprednisolone
MRI: Magnetic resonance imaging
MS: Multiple sclerosis
ON: Optic neuritis
PLEX: Plasma exchange
RRMS: Relapsing-remitting multiple sclerosis
SD: Standard deviation
SPMS: Secondary-progressive multiple sclerosis
VA: Visual acuity
